# Staff experiences of person-centred care and quality of life in nursing homes as influenced by leadership and work situation: a qualitative study

**DOI:** 10.1186/s12877-026-08594-1

**Published:** 2026-10-30

**Authors:** Håkan Eldskott, Marie Lindkvist, Johan Niklasson, Annica Backman

**Affiliations:** 1 Department of Nursing, Umeå University, Umeå, SE-901 87 Sweden; 2 Department of Epidemiology and Global Health, Umeå University, Umeå, Sweden; 3 Department of Community Medicine and Rehabilitation, Geriatric Medicine, Sunderby Research Unit, Umeå University, Umeå, Sweden

**Keywords:** Person-centred care, Quality of life, Leadership, Nursing home, Work condition.

## Abstract

**Background:**

PCC is widely recognised as an important approach to supporting QoL in nursing homes. However, staff’s ability to provide PCC may be influenced by leadership and working conditions. There is still limited knowledge of how staff themselves experience performing PCC to support residents’ QoL in everyday nursing home care. The aim of this study was to explore staff experiences of providing PCC to support resident QoL and how their provision of PCC is influenced by leadership and staff working conditions.

**Methods:**

A qualitative study design was used. Fifteen semi-structured interviews were conducted with 10 enrolled nurses and five nursing assistants working in nursing homes in four Swedish municipalities between February and May 2024. Data were analysed using qualitative conventional content analysis.

**Results:**

An overarching theme, *Creating everyday meaning despite challenges*, was supported by three categories: *Building human relationships in care*, *Connecting through communication and teamwork*, and *Overcoming obstacles for person-centred care*. Meaningful relationships, attentiveness and tailored support were described as central to providing PCC and supporting residents’ QoL. Communication and teamwork enabled staff to understand and respond to residents’ individual needs, while leadership contributed by supporting staff and facilitating teamwork. Organisational barriers, including staffing shortages, time pressure, communication difficulties and limited leadership support, constrained staff members’ ability to consistently provide PCC and support residents’ QoL.

**Conclusions:**

Staff described a strong intrinsic commitment to providing PCC that supports residents’ QoL. However, their ability to do so was shaped by leadership and organisational conditions. Enabling PCC in nursing homes therefore requires not only staff commitment, but also supportive leadership, adequate staffing and work environments that allow time, communication and continuity in care.

**Clinical trial number:**

Not applicable.

**Supplementary Information:**

The online version contains supplementary material available at https://doi.org/10.1186/s12877-026-08594-1.

## Background

Sweden’s population is aging. In 2020, over 2.6 million Swedes were aged 60 and older – a number expected to exceed 3 million within a decade. At age 60, women have an average life expectancy of 26 years, 6.5 of which are spent with reduced functional capacity, while men have an average of 23 years, with 5.1 years affected by similar limitations [1]. Consequently, it is reasonable to anticipate an increase in the need for support and assistance for older people. Nursing homes in Sweden provide 24-hour care and support for people aged 65 and older, with a core focus on ensuring a high quality of life (QoL) for their residents [1]. QoL encompasses various aspects, including physical, emotional, social, and psychological well-being

[2]. Aging itself is associated with a decline in QoL, making it essential for nursing homes to implement preventative efforts to mitigate or delay this decline [3]. Research shows that residents in nursing homes often experience reduced QoL due to factors such as cognitive impairment, neuropsychiatric symptoms, pain, depression, agitation, and decreased ability for activities of daily living (ADL) [4–7]. As a result, those living in nursing homes are at a heightened risk of lower QoL [3].

PCC is particularly relevant in nursing home care, where many residents live with dementia or cognitive impairment and may have complex care needs, including behavioural and psychological symptoms [8]. PCC emphasises tailoring care to individual preferences, needs and values while promoting dignity, autonomy and QoL for residents [9]. Person-centred approaches have also been shown to reduce agitation among nursing home residents with dementia [10], illustrating how care tailored to the individual may contribute to residents’ wellbeing. Research suggests an association between PCC approaches and the promotion of residents’ QoL, emphasising the importance of tailoring care to meet PCC criteria [11]. Thus, understanding what supports or obstructs the practice of PCC in daily care is essential for developing approaches that uphold its principles and support its further integration into care practices.

Research has shown that PCC in nursing homes may be significantly shaped by leadership engagement, staff support, and facility adaptations, all of which may influence staff members’ ability to provide appropriate and responsive care to residents with complex needs[12]. This means that staff members, including enrolled nurses (ENs) and nursing assistants (NAs), along with their managers providing leadership and guidance, collectively bear a crucial responsibility for ensuring quality care for residents in nursing homes. Their interactions and relationships with residents can significantly impact the residents’ overall wellbeing and QoL [13]. It has been shown that leaders can motivate and inspire high standards of care that align with the organisation’s mission, while inadequate leadership can result in low staff morale, increased turnover, and less effective care practices [14]. Studies have shown that a supportive organisational environment is associated with stronger PCC practices, as well as higher job satisfaction and affective commitment and lower turnover intention among staff [15]. Additionally, leadership and management within nursing homes play essential parts in creating a supportive work environment, ensuring adequate staffing levels, and promoting a culture of PCC [12]. Previous research has explored staff perspectives on PCC in nursing homes, including challenges related to its implementation and maintenance across different care settings [16]. Although PCC is associated with improved QoL and potentially better outcomes for residents, there are challenges in aligning leadership, staffing levels, and support systems to consistently foster a PCC culture [16]. Furthermore, less is known about how organisational conditions influence staff’s ability to provide PCC in ways that support residents’ QoL. This study seeks to address these knowledge gaps by examining how leadership and work conditions in nursing homes influence staff’s ability to practice PCC effectively, ultimately supporting the QoL and wellbeing of older adults in residential care.

### Aim

The aim of this study was to explore staff experiences of providing PCC to support resident QoL and how their provision of PCC is influenced by leadership and staff working conditions.

### Context

Nursing homes in Sweden provide 24-hour care for adults over 65 years of age with extensive health and care needs. The care team typically includes registered nurses (RNs), Ens, and NAs. RNs are responsible for nursing leadership and oversight of care practices, while the nursing home manager oversees the daily management of ENs and NAs. Nursing home managers also hold employer responsibility, handling staffing, scheduling, overall planning, and care quality. In this study, when referring to ‘leadership’, we specifically refer to the role of nursing home managers.

## Methods

### Design

A qualitative interview study was conducted to explore direct care staff experiences of providing PCC to support residents’ QoL. The study was designed using a conventional qualitative content analysis approach[17]. This study used the Consolidated Criteria for Reporting Qualitative Research (COREQ) [18] (Supplementary file 1).

### Sampling and data collection

The participants invited to this study were ENs and NAs working in nursing homes. The research was conducted across four municipalities in northern Sweden. Approval for the study was first obtained from the director of social services of these municipalities. Once approved, we reached out to nursing home managers at the nursing homes, who informed their staff about the study using the information we had provided. Staff interested in participating registered their interest, and the managers coordinated the scheduling of interviews during their regular working hours. The inclusion criteria were fluency in Swedish and being employed as regular staff at the nursing home, excluding temporary or on-call workers. The interviews took place at the interviewees’ workplaces, in quiet designated areas such as offices or meeting rooms, where privacy could be ensured.

The interviews were conducted by HB (PhD candidate, male) and AB (associate professor, female). HB had no prior experience in conducting qualitative research but had completed a doctoral course in qualitative methods. AB had previous experience in qualitative research. Both interviewers were familiar with qualitative interviewing techniques, ensuring a structured and ethical approach to data collection.

No prior relationship was established between the interviewers and participants before the study commenced. Participants were informed about the research aims and the general purpose of the study but were not provided with personal details about the interviewers’ backgrounds, goals, or specific assumptions. Both interviewers maintained a neutral stance during the interviews to minimize potential bias and ensure that participants’ perspectives were fully explored.

Interviews were conducted using a semi-structured guide, covering topics such as daily work routines, perceptions of PCC and strategies for promoting residents’ health and QoL in nursing homes, work conditions, leadership qualities, and the support provided. Examples of the questions asked included: ‘Can you describe a situation where you provided or witnessed person-centred care in practice? If yes, how did this affect the resident?’, ‘How would you describe your work environment and working conditions at the nursing home?’, and ‘What support does the leadership provide to promote person-centred care?’. The interview guide was developed by the research team for the purpose of this study and is provided as Supplementary file 1. Each interview was audio-recorded and transcribed verbatim, with identifying information omitted. Brief notes were taken during the interviews to support the interview process, for example, to identify issues to follow up with additional questions. These notes were not included as data or used in the analysis. Interview durations ranged from 28 to 74 min, with a total of 12 h and 54 min of recorded material. The median interview length was 52 min, and the mean length was 51 min. Interviews were conducted between February 21 and May 8, 2024. No repeat interviews were conducted; each participant was interviewed once. Transcripts were not returned to participants for comments or verification, as participant validation was not part of the study design. Data collection ended when no new information emerged.

### Participants

The study included a total of 15 participants, four men and 11 women, ranging in age from 22 to 63 years, with a mean age of 43.9 years and a median of 48 years. Their professional experience varied widely, spanning 1–32 years, with a mean of 15.8 years and a median of 13 years. In terms of educational background, 10 participants were trained ENs and five were NAs. The participants came from various national backgrounds, with eight being born in Sweden and seven born in countries outside of the Nordic region. No participants dropped out.

### Data analysis

The data were analysed using conventional qualitative content analysis as described by Hsieh and Shannon[17]. This approach was chosen to address the study aim and was considered appropriate because it allows categories to be developed inductively from the data, without relying on a predefined coding framework or quantification of the data. After the first interview was transcribed by the researchers to ensure consistency with the study’s objectives, the remaining interviews were transcribed by a professional transcription service. MAXQDA 24 was used to organise and manage the coding process, allowing for the systematic identification and analysis of codes across transcripts.

The initial coding involved multiple readings of the transcripts to capture key patterns and themes related to the aim. Codes were then grouped and iteratively refined to form broader categories, with HB and AB frequently revisiting the data to confirm the relevance and alignment of each code. This iterative, back-and-forth puzzle-building approach enabled a nuanced understanding of the material. The research team worked closely together throughout the analysis, discussing and refining codes and categories to ensure a balanced and thorough interpretation. This collaborative approach, along with regular discussions within the research team, ensured that the final categories reflected the aim.

### Ethical considerations

Permission to conduct the study was granted by the Swedish Ethical Review Board (No. 2023-06803-01). Participants received both verbal and written information about the study and provided written informed consent. To address potential discomfort during the interview, a safe and supportive atmosphere was prioritised when performing the interviews. By being approachable, respectful, and ensuring privacy throughout each session, the interviewers encouraged participants to share openly. Participants were assured of voluntary participation and the option to end the interview at any time. Confidentiality was addressed by removing identifying details such as names of workplaces. Interviews were securely stored with restricted access Table 

## Findings

The analysis resulted in one overarching theme, three categories and eleven subcategories, as presented in Table 1.

**Table 1:** outlines the main theme, categories, and sub-categories captured from the interviews

Main theme	Category	Subcategory
**Creating everyday meaning despite challenges**	**Building human relationships in care**	*Empowering through leadership* *Sensing purpose through connection* *Shaping care for each resident* *Building trust*,* joy and a sense of security*
	**Connecting through communication and teamwork**	*Striving to know the resident* *Fostering professionalism and team effort* *Ensuring structured and tailored care*
	**Overcoming obstacles for PCC**	*Desiring more leadership support* *Struggling with communication barriers* *Managing internal expectations* *Navigating staffing and time constraints*

### Creating everyday meaning despite challenges

The overarching theme highlights how the staff’s actions and practices inherently reflected both PCC and QoL principles through their caregiving. Rather than focusing on the conceptual distinction between PCC and QoL, participants described how their day-to-day efforts grounded in empathy and personal values aimed at improving residents’ QoL through tailored PCC practices. By tailoring care to individual needs, showing attentiveness and fostering dignity, staff naturally embodied the principles of PCC without necessarily labelling them as such. Leadership support was experienced as essential for providing PCC in a strained work environment. These findings suggest that leadership and supportive work environments play a crucial role in enabling or hindering these practice efforts to enhance residents’ QoL.

### Building human relationships in care

Participants described how interactions between staff and residents played a pivotal role in delivering PCC to enhance residents’ QoL. Staff emphasised the empowering effect of leadership, noting that clear guidance and support helped them feel more confident in their roles. Meaningful connections with residents allowed participants to sense a deeper purpose in their work, which was seen as crucial for providing tailored care to meet individual needs. These interactions also fostered an environment of trust, joy, and security, which were highlighted as central to creating comfort and emotional wellbeing for residents.

#### Empowering through leadership

Leadership emerged as an important factor in empowering staff members’ ability to provide PCC. Staff described feeling more empowered and confident to take initiative, collaborate with colleagues, and adapt care to residents’ individual needs when leaders were supportive, approachable, and present in everyday care. Participants particularly appreciated regular check-ins with leaders to discuss concerns and receive encouragement, often informally during coffee breaks or brief pauses in the workday. It was noted that these moments provided a relaxed setting for open dialogue, allowing staff to feel seen and supported in their roles. Participants commented that leaders who actively engaged in daily care tasks, such as assisting with residents’ personal care or organising daily schedules, demonstrated a hands-on commitment that fostered a deeper understanding of the day-to-day demands on staff. Participants also said that this involvement allowed leaders to make realistic adjustments to routines, which staff perceived as both supportive and grounded in the realities of caregiving. Leaders who balanced administrative responsibilities with visible participation in caregiving were perceived more favourably by staff, contributing to a more collaborative atmosphere. As one participant noted,

It’s important that the manager is involved and sees us; it gives us confidence.

[Participant 1]

This type of leadership was described as supporting staff morale and their ability to adapt care to residents’ individual needs and preferences. Empowered staff reported feeling more confident to take initiative, collaborate with colleagues and adjust care to better meet the individual needs of residents.

#### Sensing purpose through connection

The participants commented that forming meaningful connections with residents was essential for providing both PCC and supporting residents’ QoL, as it fostered a sense of purpose among caregivers. They described how these connections, built through daily interactions, contributed to the emotional wellbeing of the residents. This process often involved simple yet powerful actions, such as making time to talk, offering a reassuring smile, or providing gentle physical contact. These small gestures were described as a key to creating trust with the residents. Participants also reflected on the mutual emotional benefit of these connections:

When I provide quality of life to the older people, I experience it too, automatically.

[Participant 5]

Through these meaningful interactions, participants described feeling a strong sense of fulfilment, and purpose in their work. The emotional connection with residents was described as mutually rewarding, contributing not only to residents’ everyday experiences but also to staff members’ own sense of meaning and motivation in their work.

#### Shaping the care around each resident

Participants emphasised that enabling residents to make their own decisions, particularly regarding their daily routines, was central to maintaining their dignity and independence. Allowing residents to exercise control over aspects of their own care – such as choosing when to wake up or how to manage their personal hygiene – was described as a way of ensuring that the care remained respectful and aligned with the residents’ desires. The importance of honouring these choices was highlighted.

It’s the best way to maintain their quality of life – letting them make decisions about themselves.

[Participant 6]

Participants also focused on supporting residents with tasks that could be challenging due to the residents’ unfamiliarity with modern technology, such as using a mobile phone. Many older adults struggled with operating smartphones or navigating apps, which could lead to feelings of frustration or isolation. Staff stepped in to help residents make phone calls, send messages or use video call functions to stay connected with family and friends. These small acts of assistance were described as not only easing the practical challenges faced by residents but also helping bridge the gap between the residents and the fast-evolving digital world. By providing this type of support, the staff considered that they fostered a sense of connection and security, which could contribute to a more PCC experience and higher QoL.

#### Fostering trust, joy and a sense of security in care

Fostering trust, joy and a sense of security were described as being at the heart of providing PCC for in ways that supported residents’ QoL. Participants emphasised that trust was built through small, consistent actions that reassured residents and helped them feel secure. Whether through a calm, patient approach during daily care tasks or by being emotionally present, the staff strove to make the residents feel respected and valued. This sense of security was often tied to creating a calm and peaceful environment where the residents were never rushed or made to feel uncomfortable. This was achieved, for example, by staff making an effort to sit down with residents for calm conversations without rushing through tasks, which gave residents the opportunity to share their thoughts and feel truly heard. Participants also highlighted the importance of joy in the daily lives of residents. Activities such as listening to music, sharing a laugh or joking with residents were described as creating shared moments of joy between staff and residents. Participants described these interactions as strengthening emotional bonds between staff and residents, while helping residents feel more at ease and secure.

It’s so little that’s needed for them to smile, feel safe, and calm.

[Participant 5]

The role of respect in maintaining trust, joy and a sense of security was another recurring subject. Staff emphasised the need to treat residents with dignity, always addressing them by name and respecting their wishes. By doing so, staff ensured that residents felt in control of their lives, even when facing cognitive or physical limitations. This approach was described as helping to preserve residents’ sense of identity and independence, reinforcing the trust between staff and residents. By focusing on trust, joy and a sense of security, staff could create a care environment where residents not only felt safe but also experienced moments of happiness and peace, which in turn strengthened the emotional bonds between residents and staff.

### Connecting through communication and teamwork

It was highlighted that providing PCC for QoL relied on the staff’s ability to gather relevant information and adapt care strategies. Participants emphasised the importance of staying informed about residents’ needs, strengthening professionalism and teamwork to address challenges, and using individualised care plans that reflected the unique preferences and conditions of each resident. These adaptive practices were seen as crucial for delivering PCC.

#### Striving to know the resident

The process of gathering individualised information was highlighted as a fundamental aspect of delivering PCC for QoL. Rather than applying a one-size-fits-all approach, staff members emphasised the importance of deeply understanding each resident’s unique needs, preferences and daily habits. This involved not only asking the right questions but also being observant and attuned to the subtleties of non-verbal communication. Participants stressed that the key was to listen carefully, observe body language and understand behaviours to recognise what a resident might need, even when they could not express it verbally.

Participants shared how they gathered insights through ongoing conversations and careful attention to the details of daily life, from learning how a resident prefers to start the day to noticing small cues that indicated discomfort or contentment. As one participant explained,

You really have to take the time to get to know them, to be patient and listen. It’s the little things that matter.

[Participant 3]

This approach was described as ensuring that the care provided continuously adapted to reflect each resident’s evolving needs, contributing to a more meaningful and responsive caregiving experience.

#### Fostering professionalism and team effort

Participants described that maintaining professionalism while managing the emotional strain of caregiving was a vital skill for staff in nursing homes. Many participants spoke of the importance of leaving personal stress behind when interacting with residents, allowing staff to fully focus on the residents’ wellbeing. This ability to compartmentalise emotions was seen as essential for delivering PCC for QoL, particularly on demanding days.

In addition to individual professionalism, teamwork was consistently emphasised as crucial for providing care that met residents’ needs. Participants described how working together created a supportive environment where they could rely on each other during challenging shifts. This sense of collaboration not only helped to reduce stress but also ensured that residents received the attention and care they deserved. This collaborative approach was described as important:

We are all working toward the same goal, that the residents here should feel well.

[Participant 8]

This unified effort allowed staff to balance their responsibilities more effectively, knowing that their colleagues were there to offer support when needed. The combination of professionalism and teamwork gave staff a sense of fulfilment, as they felt that their efforts contributed to enhancing the residents’ QoL and making a meaningful difference in their own work.

#### Ensuring structured and tailored care

Developing and using individualised care plans were described as a crucial aspect of delivering PCC. These plans were said to systematically incorporate residents’ preferences and daily routines, ensuring that care was structured and tailored to their specific needs. When residents first moved into the facility, participants reported gathering detailed knowledge about the new residents’ backgrounds, daily routines, personal habits and preferences. This knowledge was provided by the residents themselves, as well as by family members and previous caregivers. Such information was described as helpful for understanding daily routines and personal habits.

Participants explained that care plans were regularly reviewed during weekly team meetings, typically involving registered nurses, enrolled nurses, the resident’s primary caregiver, the nursing home manager and, when relevant, allied health professionals, to identify changes in residents’ conditions and consider whether the care plan needed to be updated.

We have team meetings every week to see if there have been any changes, to make sure we are considering everything.

[Participant 4]

Despite the efforts to maintain these plans, participants mentioned facing practical challenges in ensuring that all team members consistently followed and updated the plans, which sometimes led to inconsistencies in care delivery. To overcome such challenges, participants explained that a primary caregiver was often assigned to each resident, ensuring that the care plans were followed and regularly updated, which helped maintain continuity of PCC and subsequently QoL.

### Overcoming obstacles to PCC

Several obstacles hindered the consistent application of PCC in nursing homes, ultimately affecting residents’ QoL. Participants cited inadequate leadership support as a barrier, particularly when frequent leadership changes created uncertainty about routines, staff concerns were not adequately addressed, or staffing shortages remained unresolved. Communication barriers both among staff members and between staff and residents – also emerged as a critical issue, complicating efforts to fully understand and meet residents’ needs. Internal expectations, including personal and professional pressures, further added to the strain on staff, while staffing shortages and time constraints made it increasingly difficult to balance the demands of daily care with PCC approaches.

#### Desiring more leadership support

Staff highlighted a recurring issue related to leadership support, which was a source of frustration for many staff members. Low leadership stability due to turnover was described as not only impacting the day-to-day workflow but also influencing the care provided to residents. For example, participants mentioned that, with each leadership change, new guidelines were introduced without adequate time for staff to adapt, creating confusion and inconsistency in how care was delivered to residents. Participants described how financial priorities could overshadow the wellbeing of staff and residents, for example, when resource constraints affected the number of staff, hindering the ability to take the resident for a walk.

It’s always about the money, money, money … I get that there are budgets, but it’s people we’re dealing with.

[Participant 7]

When staff felt that their concerns were not adequately addressed by leaders, they described consequences for both their own wellbeing and the care they were able to provide. For example, when staffing shortages remained unresolved, staff were often stretched thin, leading to delays in care and making it harder to attend to residents’ individual needs. Additionally, participants expressed that leaders sometimes seemed disconnected from the realities of daily care. For example, when leaders frequently overlooked staff input, the staff members felt unsupported and less motivated, which could impact their ability to fully implement PCC principles and provide tailored care for each resident.

#### Struggling with communication barriers in care

Participants commented that communication barriers, especially those related to limited proficiency in Swedish, posed challenges to providing effective care in nursing homes. Misunderstandings between staff and residents, as well as among colleagues, were common and often led to stress and frustration. Staff reported that these communication difficulties hindered their ability to fully understand residents’ needs, with possible impact on the quality of PCC. Language issues were cited as a major obstacle, with staff members struggling to communicate effectively with other staff members who did not speak Swedish fluently.

Efforts such as Swedish language courses were introduced to mitigate these challenges, but several participants said that they felt that these initiatives alone were insufficient to address the barriers. Some participants shared their own proactive approach to overcoming language difficulties, describing how they took personal initiative to improve their Swedish after receiving feedback from a resident:

A resident thought I was bad at Swedish, so I took books home and studied, and I got much better.

[Participant 2]

Participants emphasised their individual commitment to overcoming communication barriers, demonstrating the importance of continuous learning and adaptation in care work. Despite these individual efforts, they pointed out a broader need for clear and consistent communication within the care team, stressing that collaboration and open dialogue were essential for building trust and ensuring high QoL for residents.

#### Managing internal expectations

Participants described how their dedication to providing high-quality care often stemmed from a strong personal sense of responsibility and empathy. This internal drive was rooted in a deep sense of responsibility and empathy, with staff members expressing a personal commitment to providing care that was as compassionate and as attentive as possible. However, they found that balancing these high expectations with the realities of their roles often left them feeling inadequate. The emotional strain of not being able to meet both their own standards and the residents’ needs was a recurring theme, leading to feelings of stress and frustration. Participants expressed a deep personal investment in their work, describing how their sense of fulfilment was closely tied to their ability to provide attentive and individualised care to residents. This emotional connection to their role meant that falling short of their own high standards left them with a lingering sense of dissatisfaction:

If I don’t feel like I’ve done a good job, it feels like something is missing within me.

[Participant 4]

This personal commitment to compassionate care was described as often leading participants to internalise work outcomes, resulting in feelings of guilt when they could not offer residents the attention or QoL they felt was right. Participants reported that this internal conflict took an emotional toll, as they struggled to reconcile their dedication with the practical limitations of their work environment.

#### Navigating staffing and time constraints

Participants described staffing shortages and time constraints as ongoing challenges that made it more difficult to provide PCC and attend to residents’ individual needs. They expressed frustration over their inability to give residents the necessary attention due to the sheer pressure of managing too many tasks with too few people. This sense of rushing through the day left many staff members feeling overwhelmed and unable to maintain the high standards of personalised care they aspired to. Participants described the stress of working in an understaffed environment, where the pressure to meet various demands compromised the attention given to residents.

When we are short-staffed, I feel like we’re always running past residents who need attention because we are stressed about getting to something else.

[Participant 5]

This insufficiency of staff not only made it difficult to meet residents’ more complex care needs but also created an environment where staff said they felt constantly behind, contributing to heightened stress levels during already-demanding shifts. The challenges associated with staffing and time constraints were described as key barriers to delivering consistent PCC, further exacerbating the emotional and physical strain on staff, which in turn impacted the QoL of the residents.

## Discussion

This study explored staff experiences of providing PCC to support residents’ QoL and how leadership and working conditions influenced their provision of PCC. The findings were captured in the overarching theme *Creating everyday meaning despite challenges* and the three categories *Building human relationships in care*,* Connecting through communication and teamwork*, and *Overcoming obstacles for PCC*. The overarching theme reflects a tension running through staff members’ accounts: their efforts to create meaningful everyday experiences by knowing residents, responding to individual needs and preferences, and supporting their wellbeing were continually negotiated within the conditions of everyday nursing home care. While these efforts reflected principles of PCC, staff members’ opportunities to put them into practice were shaped by communication, teamwork, leadership and organisational conditions.

### Building human relationships in care

A key finding was that staff did not clearly differentiate between PCC and QoL in their everyday practice. Rather than focusing on theoretical distinctions, they described knowing the resident, responding to individual needs and preferences, supporting autonomy and creating meaningful interactions as part of supporting residents’ QoL. This is consistent with Davies et al. 19, whose meta-ethnography identified substantial interrelations between PCC and QoL in long-term residential care, including personalisation of care and ensuring that residents’ voices are heard. Our findings extend this understanding by showing how direct care staff described these principles as embedded in their everyday interactions with residents, rather than as the deliberate application of a formal PCC model. In this sense, PCC appeared to be reflected in what staff did in their encounters with residents, even when they did not explicitly conceptualise their practice in terms of PCC.

### Connecting through communication and teamwork

The findings further demonstrate that person-centred practice was not solely dependent on interactions between individual staff members and residents. Knowing residents and responding to their individual needs also depended on communication and collaboration among staff. This is consistent with Güney et al. 20, who identified teamwork as important for the provision of PCC in long-term residential care. Sharing knowledge about residents’ preferences, routines and changing needs may enable staff to develop a more comprehensive understanding of the resident and provide more consistent individualised care.

Conversely, communication difficulties could constrain these processes. In this study, participants particularly described difficulties related to limited proficiency in Swedish, affecting communication both between staff and residents and among colleagues. Similar challenges have been identified internationally. Huynh et al. 21, in a scoping review of migrant care workers in long-term care facilities, identified communication and language barriers as recurring challenges. In the Swedish context, Eriksson and Hjelm [22] found that nursing home residents experienced difficulties communicating with staff with limited Swedish language proficiency, including difficulties understanding and being understood. Taken together, these findings suggest that language proficiency may influence both communication within care teams and staff members’ opportunities to understand residents’ individual needs and preferences and thereby provide PCC to support resident health. This implies that nursing home organisations need to recognise and address language-related barriers, for example by providing appropriate language support and creating conditions that facilitate clear communication within care teams and with residents.

### Overcoming obstacles for PCC

Despite staff describing practices that were consistent with PCC, their ability to provide such care was constrained by organisational conditions. This points to a gap between the strong policy emphasis on PCC as a guiding approach to long-term care [23],[24] and its implementation in everyday nursing home practice. Previous research has similarly demonstrated the importance of organisational conditions for implementing PCC, including adequate staffing and time, supportive leadership, clear organisational structures and support for staff[16], [25], [26]. In our study, staff continued to describe a strong commitment to person-centred ways of working despite these constraints, while organisational conditions influenced their opportunities to put these values into practice. This distinction is important, as it suggests that strengthening PCC to support resident QoL may require more than increasing staff knowledge of PCC principles; it also seems to require organisational conditions that allow staff to act on them.

Leadership therefore represents an important part of the organisational context in which PCC is enacted. Similar to our findings, Richter et al. ^16^ emphasised leadership and organisational support as important for establishing conditions conducive to PCC. Supportive and accessible managers may facilitate staff members’ ability to respond flexibly to residents’ individual needs, whereas insufficient leadership support, frequent leadership changes and unresolved staffing difficulties may constrain these opportunities. However, responsibility for creating these conditions cannot rest solely with individual nursing home managers. Previous research has shown that managers’ ability to support staff in providing PCC is itself dependent on organisational support and resources [26]. Managers therefore need adequate organisational conditions to enable them, in turn, to support staff in providing PCC to support resident QoL.

The findings can be understood in relation to McCormack and McCance’s ^9^ Person-Centred Practice Framework. Staff accounts reflected several of the prerequisites for person-centred practice described in the framework, particularly interpersonal skills, commitment and person-centred values. However, the findings also illustrate a tension between having these prerequisites and being able to enact them in everyday care. Staff could be committed to knowing residents and responding to their individual needs and preferences, while limited time, staffing, communication and leadership support constrained their opportunities to do so. Viewed through the framework, this finding illustrates how different components of the framework intersect in everyday nursing home practice.

### Methodological consideration

One potential limitation of this study is researcher bias, as the primary researcher conducted both interviews and analysis. Hsieh and Shannon [17] emphasise the importance of maintaining objectivity in qualitative research, which we addressed by having team discussions throughout the analysis process to validate coding and interpretations, thus minimising subjective influence. The recursive coding process played a critical role in maintaining methodological rigor, as it allowed for continual refinement of categories, ensuring that they were grounded in the data. Team collaboration further supported this by enabling diverse perspectives to challenge interpretations, thereby strengthening the credibility of the findings. This approach aligns with Hsieh and Shannon’s ^17^ recommendations for inductive content analysis, particularly in balancing abstraction with fidelity to the data. The concept of transferability is central to qualitative research, referring to the degree to which findings can be applied in other contexts. In this study, thick descriptions of the organisational and leadership dynamics within nursing homes were provided to offer a foundation for assessing transferability. While specific findings, such as the influence of leadership support and communication, may resonate broadly, the unique cultural and institutional characteristics of Swedish nursing homes should be considered when applying these insights elsewhere. This study’s findings highlight universal themes such as the importance of collaboration and structured support, while acknowledging that differences in staffing models, leadership styles or care philosophies could influence their applicability. By providing detailed contextual information, this study aims to support readers in evaluating the relevance of its findings to their own settings.

The sample of 15 participants provided variation in age, professional experience, occupational role, and country of birth, with seven participants born outside the Nordic countries. However, as the interviews were conducted in Swedish, staff with limited Swedish proficiency may have been underrepresented, as the language proficiency required for an in-depth research interview may exceed that required in everyday nursing home care. This is particularly relevant as language and communication emerged as important conditions for providing PCC.

Additionally, the fact that the nursing home managers were involved in facilitating participant recruitment, as the interviews were conducted during staff’s ordinary work hours, may have caused selection bias. However, both oral and written information were provided to all staff at the facility, clearly stating that participation was voluntary, and no indications of coercion or non-voluntary participation were observed.

### Implications for practice

Supporting PCC in nursing homes requires more than individual staff commitment; it requires organisational conditions that allow staff to spend time with residents, build relationships and respond to what matters to them. Nursing home leaders should therefore prioritise sufficient time for resident interaction, accessible support for staff, effective teamwork and communication, and attention to language-related barriers. Adequate language proficiency is important for understanding residents’ needs and preferences and for coordinating individualised care, highlighting the need for organisational strategies to identify and address language barriers. Nursing home managers also require adequate organisational support and resources to create and sustain conditions that enable staff to provide PCC.

### Conclusion

Residents’ QoL and PCC were closely interconnected in staff members’ descriptions of everyday nursing home care. Staff described a strong commitment to person-centred ways of working, but their opportunities to put these values into practice were shaped by leadership and organisational conditions. Supporting PCC therefore requires both supportive leadership and organisational conditions that enable staff to translate person-centred values into everyday practice and thereby support residents’ QoL.

## Supplementary information

Below is the link to the electronic supplementary material.

Supplementary Material 1

## Data Availability

Availability of data and materialsThe datasets generated and analysed during the current study are not publicly available due to the need to protect participant confidentiality but are available from the corresponding author on reasonable request.

## Abbreviations

PCC: Person-centred care
QoL: Quality of life
ADL: Activities of daily living
EN: Enrolled nurse
NA: Nursing assistant
RN: Registered nurse
COREQ: Consolidated Criteria for Reporting Qualitative Research
