# Supplementary material for: Staff experiences of person-centred care and quality of life in nursing homes as influenced by leadership and work situation: a qualitative study
Source: BMC Geriatr. 2026 Oct 30;26:1371. doi: 10.1186/s12877-026-08594-1 (PMC13723137; doi:10.1186/s12877-026-08594-1)
Supplement: Supplementary file 1 [file 12877_2026_8594_MOESM1_ESM.docx]

**Supplementary file. Interview guide**

**Interview guide: Experiences of work, leadership and person-centred care in nursing homes**

**1. Introduction**

- Introduce myself as a researcher and explain the purpose of the study.
- Emphasise that participants’ opinions and experiences are valuable for understanding their perspectives on work situation, leadership and person-centred care.

**2. Background information**

- Ask participants to briefly describe their professional background and experience of working in nursing homes.
- Ask about their current role and responsibilities.

**3. Work environment and work situation**

- How would you describe your work environment and work situation in the nursing home?
- What factors influence your workload and level of stress?
- How do these factors affect your ability to provide quality care and support residents’ health and quality of life?
- Can you describe a typical working day? What are your tasks and responsibilities?
- Which aspects of the work environment do you experience as positive and supportive in your work? Why?
- Are there specific factors that create high workload or stress? Can you give examples?
- How do these factors influence your ability to provide quality care and support residents’ health and quality of life?

**4. Leadership and support**

- How would you describe leadership in the nursing home? What characteristics and behaviours define a good leader?
- What support and resources are provided by management to promote person-centred care?
- How does leadership and support influence your ability to support residents’ health and quality of life?
- Can you describe a situation where you experienced good leadership? What characterised that leadership?
- Can you give examples of how management support has influenced your work?
- How does leadership and support influence your ability to support residents’ health and quality of life?

**5. Experiences of person-centred care**

- How would you define person-centred care? What factors are important for achieving person-centred care in nursing homes?
- Can you describe situations where you have applied or observed person-centred care?
- What challenges do you encounter when providing person-centred care?
- Can you describe a specific situation where person-centred care was applied? How did it affect the resident’s experience?
- Are there barriers or difficulties you have encountered in providing person-centred care?

**6. Supporting health and quality of life**

- How do you contribute to supporting residents’ health and quality of life?
- What resources and tools are available to promote residents’ health and quality of life?
- What barriers or limitations do you experience in providing optimal support?
- Can you give concrete examples of actions or interventions you have implemented?
- How are available resources used in practice?
- Do you have suggestions for how identified barriers could be addressed?

**7. Reflections and closing**

- Is there anything else you would like to add or discuss regarding your experiences of your work?
